# Bacterial Natural Product Drug Discovery for New Antibiotics: Strategies for Tackling the Problem of Antibiotic Resistance by Efficient Bioprospecting

**DOI:** 10.3390/antibiotics10070842

**Published:** 2021-07-10

**Authors:** Yannik K. Schneider

**Affiliations:** Marbio, Faculty for Fisheries, Biosciences and Economy, UiT—The Arctic University of Norway, Breivika, N-9037 Tromsø, Norway; yannik.k.schneider@uit.no

**Keywords:** antibiotics, antibiotic resistance, natural products, bioprospecting, actinobacteria, cyanobacteria, myxobacteria, biosynthetic potential, secondary metabolites, drug discovery

## Abstract

The problem of antibiotic resistance has become a challenge for our public health and society; it has allowed infectious diseases to re-emerge as a risk to human health. New antibiotics that are introduced to the market face the rise of resistant pathogens after a certain period of use. The relatively fast development of resistance against some antibiotics seems to be closely linked to their microbial origin and function in nature. Antibiotics in clinical use are merely products of microorganisms or derivatives of microbial products. The evolution of these antimicrobial compounds has progressed with the evolution of the respective resistance mechanisms in microbes for billions of years. Thus, antimicrobial resistance genes are present within the environment and can be taken up by pathogens through horizontal gene transfer. Natural products from bacteria are an important source of leads for drug development, and microbial natural products have contributed the most antibiotics in current clinical use. Bioprospecting for new antibiotics is a labor-intensive task as obstacles such as redetection of known compounds and low compound yields consume significant resources. The number of bacterial isolates one can theoretically investigate for new secondary metabolites is, on the other hand, immense. Therefore, the available capacity for biodiscovery should be focused on the most promising sources for chemical novelty and bioactivity, employing the appropriate scientific tools. This can be done by first looking into under- or unexplored environments for bacterial isolates and by focusing on the promising candidates to reduce the number of subjects.

## 1. Introduction: Development of Antibiotic Resistance in *Staphylococcus aureus*. An Example for Acquisition of Resistance to Antibiotics in Clinical Use

**Figure 1:**
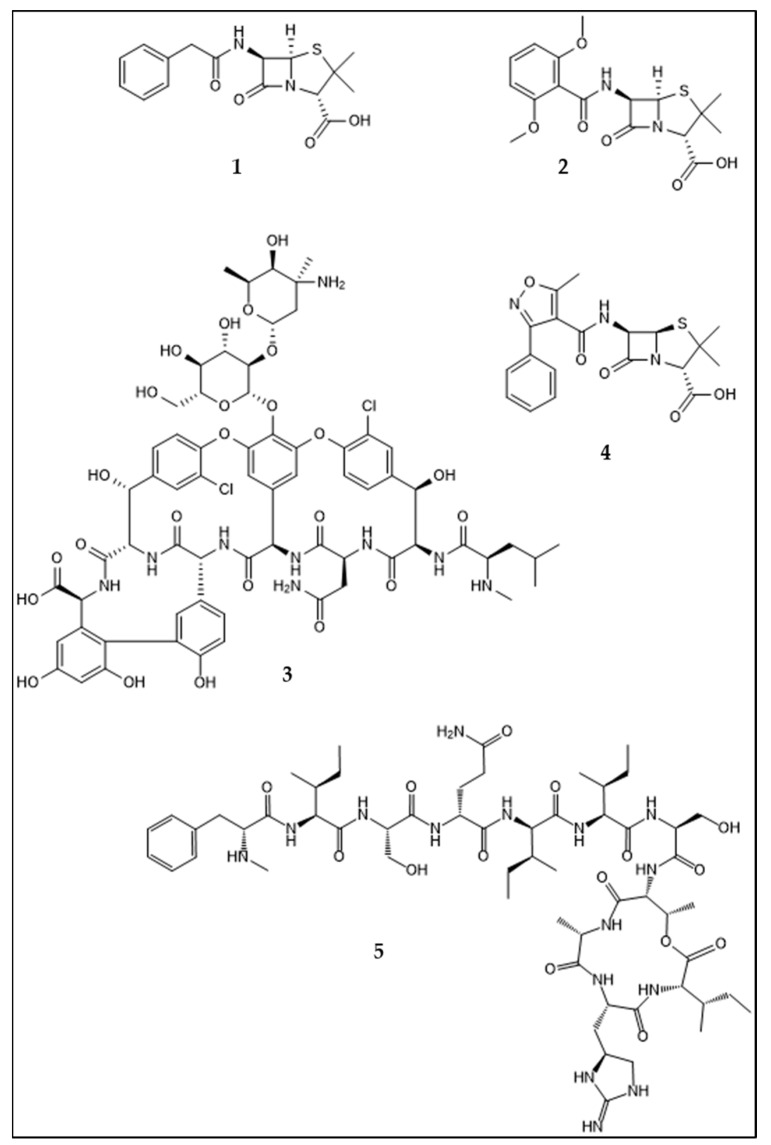
Structures of antibiotics with an anti-bacterial effect on Staphylococcus aureus/ MRSA: Penicillin G (**1**), methicillin (**2**), vancomycin, (**3**), oxacillin (**4**) and teixobactin (**5**).

**Figure 2:**
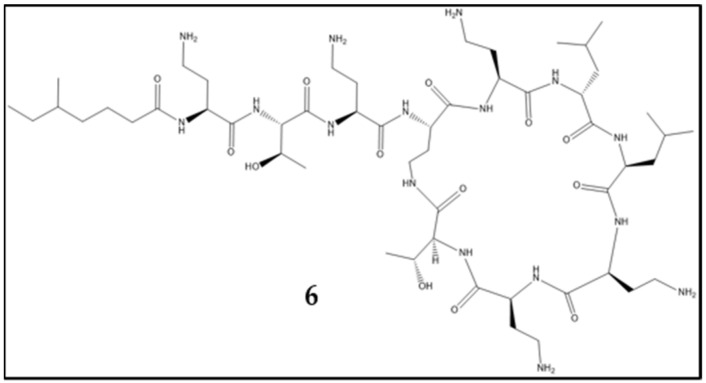
Structure of colistin (**6**).

**Figure 3:**
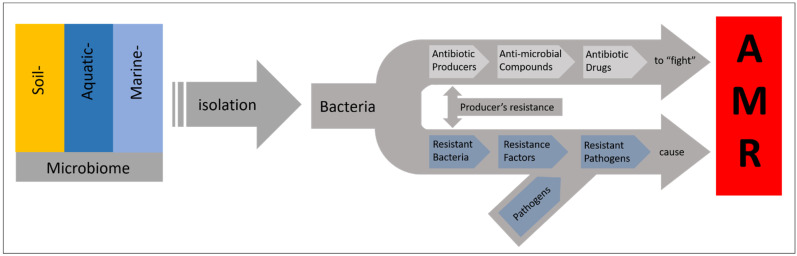
The dilemma of resistance against natural product-derived antibiotics. Antibiotic-producing bacteria exist in the same environments as antibiotic-resistant ones. Antibiotic resistance is also linked to antibiotic production by the respective producers’ resistance for “self-protection”. The resistance factors available within the environment finally contribute to the **a**nti**m**icrobial **r**esistance (AMR) of clinically relevant pathogens.

**Figure 4:**
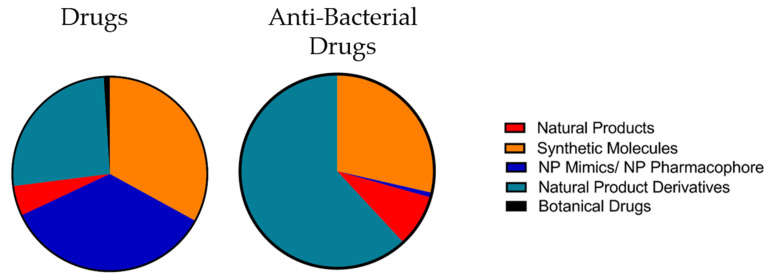
Origin of small molecular drugs from Jan. 1981 to Sept. 2019. To the left, drugs over all indications are grouped, and to the right, specifically the anti-bacterial drugs are grouped for comparison. Numbers from Newman and Cragg [45]; the classifications from the original were pooled for simplification.

After the discovery of penicillin by Alexander Fleming in 1929, synthetic sulfonamides in 1935, and streptomycin in 1944, the discovery of new antibiotics during the following decades up to the 1970s buoyed optimism that the threat of infectious diseases had been overcome [1]. However, Alexander Fleming warned already in 1945 that frequent and irresponsible use of antibiotics triggered by public demand would lead to a loss of efficacy [2]. His statement that “microbes are educated to resist penicillin” was an early warning that deserved much more attention than it actually received. More than 20 classes of antibiotics were introduced to the market between the 1940s and 1962 [1], while no new class of antibiotics reached the market between 1962 and 2000 [3]. The development of new antibiotics in the first decades of the antibiotic era kept pace with the evolving development of resistance in an “arms race” with the pathogens. This was in strong contrast to the current situation, where antibiotic resistance is considered to be a health crisis by the World Health Organization [4]. The six most problematic clinical pathogens were summarized by Louis Rice under the abbreviation “ESKAPE” bugs, namely *Enterococcus faecium*, *Staphylococcus aureus*, *Klebsiella pneumoniae*, *Acinetobacter baumanni*, *Pseudomonas aeruginosa*, and *Enterobacter* species [5]. *S. aureus*, for instance, gradually developed resistance to antibiotics as they were introduced to the marked and used in therapy. While *S. aureus* was susceptible to penicillin (Figure 1, **1**) treatment in the 1940s, 40% of the clinical isolates were penicillin-resistant by 1950, and this fraction increased to 80% by 1960 [6]. The resistance was acquired through the uptake of genes enabling the production of β-lactamases [7]. In 1959, methicillin (Figure 1, **2**) was introduced to treat penicillin-resistant *S. aureus* infections [8], and two years later the first methicillin-resistant *S. aureus* isolate was reported [9]. Consequentially glycopeptides, particularly vancomycin (Figure 1, **3**), served as a “last line of defense” against methicillin-resistant *S. aureus* (MRSA) for the next 40 years. The first resistance against vancomycin and teicoplanin was reported in 1988 in *Enterococcus faecium* [10], and nearly one decade later, in 1997, an MRSA isolate from a wound infection exhibited decreased vancomycin susceptibility [11]. Four years later, in 2002, the first vancomycin-resistant MRSA strain was discovered in a clinical setting. In contrast to isolates showing decreased susceptibility (vancomycin MIC = 4–8 µg/mL, the so-called vancomycin intermediate *S. aureus*, abr. VISA), the isolate demonstrated resistance to vancomycin (vancomycin MIC > 8 µg/mL). This was the first representative example of vancomycin-resistant *S. aureus* (VRSA) with resistance against vancomycin (MIC > 128 µg/mL) and oxacillin (Figure 1, **4**) (MIC > 16 µg/mL) [12].

## 2. Mechanisms and Acquisition of Antibiotic Resistance

The chemically heterogeneous group of antibiotics comprises several modes of action in order to possess their respective effects. The three main targets of antibiotics are the bacterial cell wall or cell wall synthesis, nucleic acid-synthesis/-replication, and protein synthesis [13]. Examples for the respective antibiotics are the β-lactam antibiotics and vancomycin-inhibiting cell-wall synthesis, rifampicin that inhibits bacterial RNA-polymerase, and tetracyclines and clindamycin-inhibiting protein synthesis at the 30S and 50S ribosome subunits, respectively [13]. The bacterial arsenal of possible resistance mechanisms, on the other hand, is diverse, too. It ranges from alteration of the target, as we will see with some examples further down, to the enzymatic degradation of the antibiotic, e.g., by β-lactamases. Another common resistance mechanism is the export of antibiotics out of the bacterial cell using efflux pumps [14].

A depictive example of the acquisition of resistance against antibiotics used for treatment of a bacterial pathogen is *S. aureus*; it also exemplifies different resistance mechanisms. Penicillin resistance is caused by β-lactamase activity [7], whereas resistance against methicillin is mediated by the *mecA* gene complex encoding penicillin binding protein 2′ (PBP2′). PBPs catalyze the crosslinking of peptidoglycan within the bacterial cell wall and are targeted by β-lactam antibiotics. The resistance factor PBP2′ shows little affinity to β-lactam antibiotics, thus mediating resistance against this class, including methicillin [15]. Resistance and tolerance against vancomycin are caused by two different mechanisms. The VISA strains show different mutations, mostly involved in cell-wall biosynthesis, and the overproduction of cell-wall material is an attribute of VISA observable by electron microscopy [16]. Vancomycin resistance in VRSA is mediated by the *vanA* operon, located within the *Tn1546* transposon. Origin of resistance is the vancomycin-resistant enterococci conjugative plasmid, which is the link between enterococcal and staphylococcal resistance [17,18]. Vancomycin resistance is mechanistically based on the exchange of an alanyl entity within the cell-wall-peptide linker into a lactyl group. Vancomycin has high affinity to the D-ala-D-ala residue, a component of lipid II, which is a building block for the bacterial cell wall and enables vancomycin to inhibit bacterial cell wall synthesis. For vancomycin resistance, the d-ala-d-ala dipeptide is altered into d-ala-d-lac, which has reduced susceptibility to vancomycin [16,19]. The different resistances acquired by *S. aureus* are examples of different mobile genetic elements conveying resistance. The β-lactamase is encoded by *blaZ* and located on plasmids [20], whereas *vecA* is located on the staphylococcal cassette chromosome [21] and the vancomycin resistance within the *Tn1546* transposon. This development may exemplify how *S. aureus* acquired resistance via different resistance mechanisms and genetic elements. The emergence of new antibiotic resistance against last-resort antibiotics is ongoing. As seen above, the location of the antibiotic resistance gene on mobile genetic elements is an important factor for their spread into other strains and genera. More recently, in 2016 a plasmid-borne resistance against colistin (Figure 2, **6**) was discovered in China, and its uptake by ESKAPE pathogens was reported in 2017 [22,23,24], providing a more contemporary example of the described problem.

## 3. The Ancient Origin of Antibiotic Resistance

After discussing the function of antibiotic resistance and its spread by horizontal gene transfer, the question of resistance origin remains. It is a common perception that antibiotic resistance has been induced via use and misuse of antibiotics by humans, thereby triggering the evolution of the molecular targets to develop resistance by mutation and selection. However, this anthropogenic scenario is just partly true [25]. Antibiotic resistance itself evolved long before the first humans appeared on Earth. Antibiotic-resistant bacteria have been isolated from Siberian permafrost sediment, dating back 3 × 10^3^ to 3 × 10^6^ years, with resistance against antibiotics such as chloramphenicol, tetracycline, and aminoglycosides [26]. In 2016, a *Paenibacillus* sp. isolate was found to carry resistance against 26 of the 40 tested antibiotics, including daptomycin, which was introduced to the market in 2003. Notably, the cave it was isolated from had been cut of from the surrounding environment for 4 × 10^6^ years [27]. In another study, with isolates from Beringian permafrost samples, it was shown that a *vanHAX* cluster, encoding for glycopeptide resistances, was clustering with genes of recent organisms, showed functional as well as structural similarity of the gene products, and was capable of mediating genuine resistance proven by heterologous expression in *E. coli* [28]. In addition to the experimental findings, structure-based phylogeny suggests that metallo-β-lactamases evolved more than 2.2 × 10^12^ years ago [29]. The structure- and sequence-based phylogeny of serine-β-lactamases suggests that they evolved around 2.2 × 10^12^ to 2.4 × 10^12^ years ago, depending on the respective class [30]. Thus, antibiotic resistance to different antibiotics was already present within the environment before they came into clinical use. Given the already existing presence, if not omnipresence, of antibiotic-resistance genes in nature and the various mechanisms of horizontal gene transfer, the fast rise of antibiotic resistance in pathogens, only a few years after exposure to clinical use of the respective antibiotic, is, in retrospect, no surprise [31]; see Figure 3 for a schematic overview. However, the uptake of resistance factors is not the only path to resistance development. Antibiotic resistance by mutation (*de novo*) and subsequent selection can be observed, for instance, in long-term antibiotic treatment of patients [32] and contributes to the problem of antibiotic resistance. The resistance against synthetic antibiotics cannot be derived from resistance genes that have co-evolved in nature [33], and is therefore a product of more recent evolutional processes that happened after the introduction of the drugs. The role of antibiotics in nature is assumed not to be primarily as antimicrobial agents; in order to possess an antibiotic effect, the concentrations within natural habitats attributable to antibiotic-producing microorganisms are mostly too low. Other functions, such as bio regulation, intercellular signaling, or quorum sensing, seem to be the original, main purpose of the molecules in their natural environment [31]. Antibiotic resistance probably co-evolved within this frame as (regulative) responses to antibiotics serving as signaling molecules [31]. Given the presence of resistance-carrying bacteria within the environment, there are many ways for pathogens to come in contact with bacteria carrying resistance genes, for example, via wastewater from hospitals reaching waterbodies such as rivers, serving as an incubator for the exchange of those resistance factors [34]. In addition to the environmental potential for antibiotic resistance, made up by the present resistances, humans cause a selective pressure. Human use and misuse of antibiotics may be coupled to the dispersion of antibiotics into the environment, which makes the aforementioned mechanisms more likely to take place or to increase in frequency. This theory is supported by the observation that resistance formation rates correlate with the consumption of antibiotics in different countries [35,36]. Additionally, the heavy use of antibiotics in agriculture may represent a problem because it contributes to the selection and spread of antibiotic resistance within the environmental bacterial community; antibiotic-resistant bacteria can be distributed together with agricultural products, and farmers may even act as vectors for resistant microbes [36]. A study of archived soil samples from the Netherlands has shown that antibiotic resistance genes present in soil have increased significantly since 1940 [37]. It is noteworthy that soil represents an important source of antibiotic resistance, and it has been shown that agricultural use of antibiotics can increase the prevalence of resistance genes in soil [25]. Because the majority of our marketed antibiotics have been isolated from soil microorganisms, the presence of the corresponding resistance elements is no surprise, taking the aforementioned roles of antibiotics in nature into account [38]. Remarkably, the ocean, as the other and even bigger part of the global environment, harbors resistance genes similar to those found within the terrestrial environment [39]. The consequences of antibiotic resistance for our healthcare systems and society are already alarming, and the problem is likely to intensify. As a review of antimicrobial resistance projects, by 2050 there will be 10^7^ deaths per annum worldwide due to antimicrobial resistance, causing global economic damage of 10^14^ USD per annum [40]. In addition to the problem of rising antibiotic resistance, the need for potent antibiotics in the clinical setting is likely to increase because of an aging population, the increasing use of immunosuppressive therapies, cancer treatments, surgeries, and the treatment of other diseases that require anti-infectives or are more likely to require them, such as, for instance, diabetes [41,42]. To conclude, the cellular mechanisms for antibiotic resistance have evolved from another geological age and were present in our environment even before humans appeared, not to speak of clinical antibiotics. However, this does not exempt us from the obligation to use antibiotics responsibly. The spread of antibiotics into the environment, their use in agriculture, and their over-prescription certainly contribute significantly to the problem of rising antibiotic resistance. The most responsible use of antibiotics will not prevent antibiotic resistance from occurring, but it will certainly reduce and delay it. While some antibiotic-resistance factors are ancient, the use and misuse of antibiotics promotes their spread and presence in pathogens as well as the evolution of new factors [14,43]. A very important factor on that pathway is the mobilization of the respective resistance-factor encoding genes on mobile genetic elements [44].

## 4. Natural Products in Drug Discovery

The discovery and investigation of natural products have delivered numerous active pharmaceutical ingredients and lead structures for pharmaceutical development. Newman and Cragg analyzed the origin of new drugs between 1981 and September 2019 [45], and within that time-span, 1394 small molecular drugs were approved. In relative ratios, purely synthetic drugs accounted for 33% of the new drugs, 35% were synthetic natural product mimics or synthetics with a natural product as pharmacophore, 5% natural products, 26% natural product derivatives, and 1% defined as botanical drugs [46]. While the number of “genuine” natural products that reached the market as unmodified molecules is relatively low, together with the natural product derivatives they account for 31% of all drugs (excluding the botanical drugs), coming close to the share of synthetic drugs. While natural products and natural product derivatives are closely related, synthetics with natural product pharmacophore or natural product mimics represent an “intermediate” group. However, it still implies that 67% of the drugs approved between 1981 and September 2019 are either directly or at least in some more or less abstract way related to the structures of natural products [46]. If we take a closer look at the data for the small molecular antibacterial drugs exclusively, we see that natural product derivatives and natural products together account for 70.6%, synthetic drugs for 28.6%, and the intermediate classes for approx. 0.8%. For a comparison, see Figure 4. The natural products, or more precisely, their structural scaffolds, seem to play a significant role in the antibiotic field. It is noteworthy that the term “antibiotics” was primarily dedicated to antibacterial compounds that were of microbial origin, whereas the term for the synthetic pendant was “antibacterials” [41]. However, this distinction faded over the course of time, and the word “antibiotic” now commonly refers to both synthetic molecules and molecules of biological origin [44].

### 4.1. Chemical Characteristics of Natural Products

The chemical properties of natural products and synthetic compounds in compound collections for drug screening were investigated and compared. Those investigations provided the insight that natural products have a lower number of nitrogen, halogen, or sulfur atoms compared to synthetic compounds. Natural products possess more chiral centers, oxygen atoms, sp^3^-hybridized bridgehead atoms, more rigid fused ring systems, and on average, a higher molecular weight [47,48]. However, when performing comparison studies between drugs, natural products, and synthetic compounds, it should be kept in mind that the drug category itself consists of the other two groups (i.e., synthetics and natural products), and the properties of one or another group are reflected by the properties of a certain share of the approved drugs [48]. Nonetheless, investigating the structural similarity between natural products and synthetic compounds from different databases and screening libraries, Henkel et al. found that 40% of the natural product structures were not represented by structures of synthetic compounds, which indicates the suitability of natural products as a source for chemical novelty [47].

In 1988, Evans et al. reported an observation of certain molecular patterns that bind more than one ligand, termed by the authors as “privileged structures”, hence providing a viable source for medicinal chemistry [49]. Natural products themselves have the intrinsic property of interacting with protein targets because they are products of biosynthetic pathways employing enzymes, and they are often ligands to protein targets/receptors [50,51]. The structural properties of natural products and their interaction with proteins evolved over millions of years and are therefore optimized for ligand-target interaction [50]. The antibiotics produced by microorganisms have, moreover, been optimized by evolutionary processes to pass cell walls and membranes of target bacteria [52]. Ganesan investigated 24 unique natural products (according to rather strict criteria) that led to an approved drug between 1970 and 2006 [53]. He found that half of them fall into what he calls the “Lipinski universe”, which means that only one of the “Rule of five” were violated. The other half falls in what he calls the “parallel universe”. For both classes, 50% of the candidates led to orally administrable drugs, and this is at first glance in conflict with the “Rule of five”. But this may be explained by Lipinski’s fifth rule: “Compound classes that are substrates for biological transporters are exceptions to the rule” [54]. Given the limited biosynthetic pathways of natural products and the unknown substrate promiscuity, active transport may account for the high bioavailability of natural products [53]. Ganesan states that “log P is the lord of the rules”, being the most important for the evaluation of bioavailability. The molecules that fall into the “Lipinski universe” have an average molecular weight of 319 Da and an average log P of 0.0, while those of the “parallel universe” have an average molecular weight of 917 Da and log P of 2.2, indicating that log P is remarkably stable despite of an almost threefold increase in molecular weight. This can be explained by incorporation of polar functional groups enabling high molecular weights while maintaining drug-like log P values. In addition, natural products may employ intramolecular H-bonds to increase permeability and undergo structural rearrangement when interacting with their target [53].

### 4.2. Suitability of Natural Products for Drug Discovery

During the 1990s the pharmaceutical industry turned away from natural product drug discovery and focused resources on high throughput screening of libraries generated by combinatorial chemistry. Those combinatorial libraries were more suitable/practical for high throughput screening and easier to generate [55,56]. However, according to János, the hit-rate when screening natural products is magnitudes higher than screening combinatorial libraries [57]. From an estimated 3 × 10^6^ to 4 × 10^6^ compounds synthesized by the pharmaceutical industry, around 0.001% became approved drugs while at the same time 0.2 to 0.3% of the ten of thousands (>5 × 10^4^) of microbial metabolites became approved drugs and another share of the same size served as lead compounds [57]. The insufficient outcome of a classical high-throughput screening for antibiotic compounds using combinatorial libraries is also reflected by a study at Glaxo Smith Kline, where 67 high-throughput screening campaigns against different antibacterial targets resulted in 16 projects leading to hits, and five of them resulted in leads. In addition to the target-based screening, three cell assay-based campaigns were executed, one of which led to three hits but not to lead identification [58].

Examining the molecular weight and polarity of antibiotics compared to drugs for other indications such as neurological diseases targeting the central nervous system, it appears that antibiotics are on average more hydrophilic and slightly larger [58]. The fact that the chemical properties of antibiotics differ may apparently indicate that their target organisms, bacteria, are different from man. One property that speaks particularly in favor of marine natural products is their higher potency compared to compounds from terrestrial origins, which is most likely a consequence of the high dilution within the marine environment, allowing only compounds with high potency to come into effect [59]. Thus, natural products of marine origin are more likely to reach the bioactivity threshold for antibiotic agents, which are minimal inhibitory concentrations of <1–10 µM for Gram-positive and 10–100 µM for Gram-negative pathogens [60]. A statistical investigation by Kong et al. revealed that marine natural products show a large share of novelty compared with natural products from terrestrial origin, but on the other hand, they show a higher hydrophobicity, which can be explained by the reduced abundance of oxygen within the marine environment [61].

## 5. Microorganisms as Producer of Natural Products and Hurdles in Bioprospecting

Most of the antibiotics approved for medical use are products of microorganisms or derived from their metabolites, as described above. Fungi and bacteria are the classical producers of antibiotics. Among the bacteria, Actinobacteria contribute the lion’s share of bioactivity and are responsible for 90% of commercial antibiotics [62]. Within the aforementioned study of Ganesan, 19 of the 24 natural products that led to approved drugs between 1981 and 2006 were products of soil microorganisms, and the remaining five compounds were of plant origin. The compounds of microbial origin split further into four produced by fungi, two produced by bacteria, and 13 compounds produced by Actinobacteria. Notably, the investigated drug classes are not limited to antibiotics, but the numbers underscore the biosynthetic potential of Actinobacteria contributing more than half of the compounds [53]. However, there are practical reasons that make the search for novel microbial natural products a difficult task. First, there is the problem of re-investigating known compounds, which can be overcome by efficient dereplication of active bacterial extracts at an early stage in the bioprospecting workflow. The work with microorganisms in biodiscovery may present other obstacles such as silent gene clusters or difficulties in the isolation and cultivation of the organisms [63]. To shed light on these silent gene clusters, different strategies have been established and utilized, such as co-cultivation or molecular biological techniques including heterologous expression or promotor insertion [64]. The bioprospecting workflow, traditionally based on “top down” methods starting with the biological and chemical characterization of the metabolites produced by an organism, has been extended by the newer “bottom up” techniques available, using genetic information to assess the biosynthetic potential of microorganisms based on bioinformatics and molecular biology [65]. Genome mining and heterologous expression enable us to detect and access silent gene clusters, whereas metagenomic techniques provide the possibility to circumvent the problem of cultivability [63,65]. The isolation and culturing of yet “uncultivable” bacteria have made substantial advances, too; techniques in the field of membrane diffusion-based cultivation, cell sorting-based cultivation, and microfluidics-based cultivation were developed and applied to culture the uncultivable majority within the bacterial realm [66]. In the field of microbiology, the OSMAC (one strain many compounds) approach and improvements in the cultivability of microorganisms enable us to produce compounds and isolate bacteria not previously accessible [67]. However, ultimately the compound has to be produced, extracted, and purified to obtain material for structure elucidation and bioassays, but genomics can serve as an indicator of where to allocate available resources to find new molecules [52]. One of the most challenging tasks after cultivating bacteria is to identify and distinct compounds that are likely to be new or likely to be known; in combination with bio testing of the respective fermentation broth or extract, this can reveal that a known antibiotic is responsible for the observed bioactivity. This working step, called “dereplication”, is mostly based on HPLC-MS analysis, with subsequent interpretation of the spectra and database searches using the elemental composition and eventually the fragment pattern of the respective analyte in order to identify it. Here, new software using machine learning has eased the identification of known molecules and the assessment of “unknowns”—for instance, Sirius for predicting and analyzing MS2 data based upon structures, circumventing the necessity of MS2 reference spectra [68]. Metabolite databases such as METLIN ease the identification of known, as well as the characterization of unknown, compounds [69]. Important to mention as a significant improvement on the metabolomics front is GNPS (Global Natural products Social Molecular Networking), an automated metabolomics networking workflow and database search platform that has made the generation of metabolic networks feasible in particular for people who are not dedicated experts in MS2 data analysis but want to make use of that technique [70]. In a similar manner, antiSMASH has eased the process of genome mining bacterial (and eukaryotic) genomes for biosynthetic gene clusters on the genomics front [71]. A recent success story on antibiotics was the discovery of teixobactin in 2015 (Figure 1, **5**), a new antibiotic produced by a bacterium that was isolated using the isolation chip approach [72,73]. The compound is effective against MRSA, *Enterococcus*, and other problematic pathogens while having a low risk of triggering resistance because of its highly conserved targets undecaprenyl pyrophosphate, lipid I, and lipid II [72,74].

## 6. Reaching out in Less Investigated Environments to Find Novel Isolates and Compounds

One research cruise, field trip, or isolation campaign can easily yield many hundreds to thousands of bacterial isolates. The bottleneck in bioprospecting bacteria is thus extraction and screening rather than isolating bacterial strains. One problem is, as mentioned above, the frequent detection of known compounds with antibiotic effects; for instance one percent of soil Actinobacteria are streptomycin producers while one in 10^7^ Actinobacteria produces daptomycin [52]. The bottom-up and top-down approaches represent in both ways a labor and cost factor that reasonably should be focused on the most promising subjects of investigation. One strategy for selecting bacteria for bioprospecting in order to gain high hit rates of chemical novelty is to sample the, to date, untapped or less sampled ecosystems. For the marine environment, the polar seas are a promising source because about 3% of marine natural products have been isolated from polar marine organisms [75]. Less than 2% of the natural products have originated from deep sea samples [76]; thus, the polar sea represents a less investigated ecosystem, substantially different from the terrestrial/soil ecosystems that historically have been the main source for antibiotic-producing microorganisms. The concrete source to obtain new isolates for a bioprospecting campaign depends, of course, on the individual scientist’s resources, experience, and facilities and can vary from certain geographical locations down to organisms as sources for isolates [77].

## 7. Selecting the Proven Prolific Producers of Natural Products–Characteristics and Indicators for the Biosynthetic Potential of Bacteria

Another strategy to enhance the antibiotic hit rate in bioprospecting is to focus on bacteria belonging to phylogenetic groups that have been shown to be frequent producers of antibiotics or bioactive compounds. Here, the Actinobacteria have served as the most important producers of active compounds to treat different diseases. In the above-mentioned study of Ganesan, Actinobacteria were the producers of more than half of the natural products that led to an approved drug. Besides the compounds that were developed into drugs, the Actinobacteria accounted by the year 2002 for approx. 53% of the discovered antibacterial compounds of microbial origin (fungi 30%, other bacteria 18%, approximate numbers) [57]. Actinobacteria are mainly soil dwelling bacteria but also present in fresh and salt water. They are Gram-positive, GC rich, and many have the ability to form mycelia and spores [78]. The most interesting property of Actinobacteria for natural product chemistry is their ability to produce a wide variety of bioactive secondary metabolites; in particular, the genus *Streptomyces* is a producer of a high number of antibiotics. There are statistical estimations that only 3% of the antibiotics produced by *Streptomyces* have been found [79]. The genomes of Actinobacteria, especially the *Streptomyces*, show a high content of biosynthetic gene clusters, in particular for non-ribosomal peptides and polyketides, which can account for more than 5% of the genome. Interestingly, some of the Actinobacteria and all *Streptomycetes* have linear genomes that are, in the case of *Streptomyces*, as large as 8–10 Mb and may contain over 20 biosynthetic gene clusters [80]. The investigation of marine Actinobacteria has already resulted in the discovery of salinosporamide A, and marine Actinobacteria are a promising source for further new, secondary metabolites [81]. Another group with high biosynthetic potential is Myxobacteria. Members of this group probably have the most complex life cycles and “behaviors” in the bacterial kingdom. The Gram-positive δ-proteobacteria are able to glide over surfaces and “hunt” other bacteria and fungi, form biofilms, or move toward nutrient sources. The ability to “hunt” other microorganisms includes also the ability to lyse them by excretion of bacteriolytic enzymes [82,83]. Moreover, they have the ability to form spores, so-called myxospores, in fruiting bodies under unfavorable conditions [83]. Their physiological complexity is reflected by their large genomes (9 to 12.5 Mb), the latter being the largest genome within the bacterial kingdom [83,84]. Myxobacterial genomes contain a high number of polyketide syntethases (PKS), non-ribosomal peptide synthetases (NRPS), and NRPS/PKS-hybrid gene clusters, and antibiotic compounds of the mentioned classes have been isolated from myxobacteria [85,86]. Although it was previously thought that myxobacteria are exclusively terrestrial organisms, halotolerant and obligate marine strains have now been reported [84]. However, the isolation and cultivation of marine myxobacteria are difficult, and this is the main reason why they are a less investigated resource [85]. Other bacterial taxa with considerable biosynthetic potential are cyanobacteria and bacilli [60,87,88], which are both genera that show in general rather complex or complex morphologies and life cycles (spore formation, specialized cells such as heterocysts in cyanobacteria, cellular organization, e.g., into filaments, etc.).

Schinke et al. reviewed antimicrobial compounds produced by marine bacteria that have been discovered from 2010 to 2015 [60]. It appears that Actinobacteria were the most prolific producers of new compounds (*n* = 27), followed by bacilli (*n* = 12) and γ-proteobacteria (*n* = 3). The compounds produced by bacilli and γ-proteobacteria were active against Gram-negative bacteria while seven of the actinobacterial products showed activity against Gram-negatives. It is noteworthy that the majority of the active Actinobacteria and bacilli were isolated from sediment [60]. In another statistical investigation by Hu et al., the authors investigated bioactive compounds (including all bioactivities such as anti-cancer, anti-inflammatory, etc.) isolated from marine organisms between 1985 and 2012 [89]. The ratio between the total number of isolated compounds to active compounds was 47.01% for Actinobacteria and 46.38% for other bacteria. Both values are significantly over the average of 28.39% for all marine macro- and microorganisms [89]. It is remarkable that the phylogenetic groups with the highest biosynthetic potential in the bacterial kingdom often show rather complex morphologies and life cycles. On top of that, it seems that large genomes are an indicator for biosynthetic potential. A study by Donadio et al. reinforced that hypothesis [90]. They investigated 223 genomes for the presence of PKS and NRPS clusters, and those clusters were not present or rare in genomes < 3 Mb [90]. In another study, Belknap et al. investigated 1110 available *Streptyomyces* genomes and found a significant positive correlation between genome size and the number of biosynthetic gene clusters per genome [91]. So, there is another, more general indicator for promising subjects for investigation that follows an inner logic because additional genes need to be encoded within the genome besides the genes for the primary metabolism of the bacteria. In addition to the genome size, I want to stress again the tendency that appears when looking at the genera mentioned above. To have a complex life cycle (sporulation and germination), but even more, to have a complex cellular morphology seems to be a potential indicator for genera to focus on during bioprospecting efforts.

## 8. Conclusions

Bacteria have contributed an important share of the medicines in clinical use. Their metabolic machinery is able to produce a wide range of secondary metabolites with a wide range of biological activities. When it comes to natural products with strong pharmaceutical activities, they often turn out to be produced by bacteria. The anti-cancer chemotherapeutic trabectedin, primarily isolated from a tunicate, is probably the product of a symbiotic bacteria [92]. Another example is tedrodotoxin, which can be found in some species of pufferfish that are well known for their toxicity. It is one of the most potent small molecular toxins accumulated in some organs of the pufferfish but produced by bacteria [93]. The products of bacteria have served as drug leads or active pharmaceutical ingredients for many therapeutic areas such as antibiotic, immunosuppressive, and anti-cancer drugs [53]. However, the field of antibiotics is somewhat different from other pharmaceutical areas. As we isolate antibiotics from the same natural environment that is the origin for potential resistances to the pathogens, it is very likely that we will face the development of antibiotic resistance for every natural-product antibiotic that comes into clinical use. This can happen via uptake of resistance factors or mutation. It is up to us to delay the process by establishing a best practice in use of antibiotics [44]. In addition to the search for new antibiotics, the “preservation” of reserve antibiotics through responsible use should be an integral part of the strategy. In order to find new antibiotic molecules as reserve antibiotics against resistant pathogens, it is in my opinion most promising to further investigate the bacterial phyla that are known to produce secondary metabolites, to select the less-investigated branches of those phyla, isolates from less-sampled or un-sampled habitats, and to look on those that are difficult to isolate and cultivate. One environment that is therefore predestined is that found in the Arctic and Antarctic waters. To me, the combination of “sampling strategies for novelty”, e.g., isolating specifically Actinobacteria from the Arctic deep sea, seems to be a reasonable approach. Natural products from bacteria are a proven source of chemical novelty, and they have led to the development of many drugs, especially in the field of antibiotics. However, by reviewing the natural products literature, one may gain the impression that combinatorial and synthetic chemistry is “outdated”, which is not the case. Keeping the numbers from Newman and Cragg in mind, the pure synthetics made up one-third of the small molecular drugs for all indications. The toolset for finding new molecules in bioprospecting pipelines has expanded through recent and ongoing developments in bioinformatics, which provides tools to identify new biosynthetic gene clusters and predict what kind of molecules they produce. Methods in molecular biology that enable sequencing, heterologous expression, and production of metabolites are also available. These techniques provide a powerful addition to the classical bioprospecting workflow based on bioassay-guided compound isolation. The isolation of the bioactive compounds still has a crucial role, as it is required to finally identify the active compound(s), determine its bioactivity, and obtain material for structure elucidation. The bioactivity screening of extracts and fractions frequently leads to the rediscovery of known compounds and false positives via unspecific bioactivity. In our lab, we frequently obtain hits in anti-cancer and antimicrobial assays caused by lipids such as rhamnolipids [94]. Here, HPLC-MS/MS guided dereplication provides an important tool to exclude the known or trivial bioactivities. The combination of retention time, UV/Vis spectrum, mass, and fragment masses sometimes allows an identification of known bioactive compounds with high certainty, and sometimes it allows at its best an “educated guess”. However, it should be kept in mind that the identification of a known active compound and subsequent termination of the investigation of an extract carry the risk of missing other, less abundant active compounds. As mentioned above, all the possible workflows and tools in bioprospecting represent an economic and personal effort, and one can easily get lost in trying to deal with the emerging number of samples, e.g., when employing the OSMAC approach. The key to efficient bioprospecting in my opinion is finally to focus on the investigation of promising bacterial phyla, but to avoid reinvestigation of known compounds. Additional indicators to consider for promising isolates among a given collection may be the genome size of bacteria and a complex morphology and/or life cycle.

## References

[1] Coates A., Hu Y., Bax R., Page C. (2002). The future challenges facing the development of new antimicrobial drugs. Nat. Rev. Drug Discov..

[2] Fleming A. (1945). Penicillin’s finder assays its future. N. Y. Times.

[3] Bax R.P., Anderson R., Crew J., Fletcher P., Johnson T., Kaplan E., Knaus B., Kristinsson K., Malek M., Strandberg L. (1998). Antibiotic resistance—What can we do?. Nat. Med..

[4] World Health Organization (2012). The Evolving Threat of Antimicrobial Resistance: Options for Actions.

[5] Rice L.B. (2008). Federal funding for the study of antimicrobial resistance in nosocomial pathogens: No ESKAPE. J. Infect. Dis..

[6] Chambers H.F. (2001). The changing epidemiology of *Staphylococcus aureus*?. Emerg. Infect. Dis..

[7] Murray B.E., Moellering R.C. (1978). Patterns and mechanisms of antibiotic resistance. Med. Clin. N. Am..

[8] Tsubakishita S., Kuwahara-Arai K., Sasaki T., Hiramatsu K. (2010). Origin and Molecular Evolution of the Determinant of Methicillin Resistance in Staphylococci. Antimicrob. Agents Chemother..

[9] Jevons M.P., Rolinson G.N., Knox R. (1961). “Celbenin”-Resistant Staphylococci. Br. Med. J..

[10] Leclercq R., Derlot E., Duval J., Courvalin P. (1988). Plasmid-mediated resistance to vancomycin and teicoplanin in *Enterococcus faecium*. N. Engl. J. Med..

[11] Hiramatsu K., Hanaki H., Ino T., Yabuta K., Oguri T., Tenover F.C. (1997). Methicillin-resistant *Staphylococcus aureus* clinical strain with reduced vancomycin susceptibility. J. Antimicrob. Chemother..

[12] Sievert D.M., Boulton M.L., Stoltman G., Johnson D., Stobierski M.G., Downes F.P., Somsel P.A., Rudrik J.T., Brown W., Hafeez W. (2002). *Staphylococcus aureus* resistant to vancomycin-United States. JAMA J. Am. Med. Assoc..

[13] Kapoor G., Saigal S., Elongavan A. (2017). Action and resistance mechanisms of antibiotics: A guide for clinicians. J. Anaesthesiol. Clin. Pharm..

[14] Aslam B., Wang W., Arshad M.I., Khurshid M., Muzammil S., Rasool M.H., Nisar M.A., Alvi R.F., Aslam M.A., Qamar M.U. (2018). Antibiotic resistance: A rundown of a global crisis. Infect. Drug Resist..

[15] Hiramatsu K., Cui L., Kuroda M., Ito T. (2001). The emergence and evolution of methicillin-resistant *Staphylococcus aureus*. Trends Microbiol..

[16] Gardete S., Tomasz A. (2014). Mechanisms of vancomycin resistance in *Staphylococcus aureus*. J. Clin. Investig..

[17] Brandi M., Limbago A.J., Kallen W.Z., Eggers P., McDougal L.K., Albrechta V.S. (2014). Report of the 13th Vancomycin-Resistant Staphylococcus aureus Isolate from the United States. J. Clin. Microbiol..

[18] Arthur M., Molinas C., Depardieu F., Courvalin P. (1993). Characterization of Tn1546, a Tn3-related transposon conferring glycopeptide resistance by synthesis of depsipeptide peptidoglycan precursors in Enterococcus faecium BM4147. J. Bacteriol..

[19] Arthur M., Courvalin P. (1993). Genetics and mechanisms of glycopeptide resistance in enterococci. Antimicrob. Agents Chemother..

[20] Olsen J.E., Christensen H., Aarestrup F.M. (2006). Diversity and evolution of blaZ from Staphylococcus aureus and coagulase-negative staphylococci. J. Antimicrob. Chemother..

[21] Zhang Y., Agidi S., Lejeune J.T. (2009). Diversity of staphylococcal cassette chromosome in coagulase-negative staphylococci from animal sources. J. Appl. Microbiol..

[22] Liu Y.Y., Wang Y., Walsh T.R., Yi L.X., Zhang R., Spencer J., Doi Y., Tian G., Dong B., Huang X. (2016). Emergence of plasmid-mediated colistin resistance mechanism MCR-1 in animals and human beings in China: A microbiological and molecular biological study. Lancet Infect. Dis..

[23] Zhao F., Feng Y., Lü X., McNally A., Zong Z. (2017). IncP Plasmid Carrying Colistin Resistance Gene mcr-1 in Klebsiella pneumoniae from Hospital Sewage. Antimicrob. Agents Chemother..

[24] Carroll L.M., Gaballa A., Guldimann C., Sullivan G., Henderson L.O., Wiedmann M. (2019). Identification of Novel Mobilized Colistin Resistance Gene mcr-9 in a Multidrug-Resistant, Colistin-Susceptible Salmonella enterica Serotype Typhimurium Isolate. mBio.

[25] Kozhevin P., Vinogradova K., Bulgakova V. (2013). The soil antibiotic resistome. Mosc. Univ. Soil Sci. Bull..

[26] Mindlin S., Soina V., Petrova M., Gorlenko Z. (2008). Isolation of antibiotic resistance bacterial strains from Eastern Siberia permafrost sediments. Russ. J. Genet..

[27] Pawlowski A.C., Wang W., Koteva K., Barton H.A., McArthur A.G., Wright G.D. (2016). A diverse intrinsic antibiotic resistome from a cave bacterium. Nat. Commun..

[28] D’Costa V.M., King C.E., Kalan L., Morar M., Sung W.W.L., Schwarz C., Froese D., Zazula G., Calmels F., Debruyne R. (2011). Antibiotic resistance is ancient. Nature.

[29] Garau G., Di Guilmi A.M., Hall B.G. (2005). Structure-Based Phylogeny of the Metallo-β-Lactamases. Antimicrob. Agents Chemother..

[30] Hall B.G., Barlow M. (2004). Evolution of the serine β-lactamases: Past, present and future. Drug Resist. Updates.

[31] Aminov R.I. (2009). The role of antibiotics and antibiotic resistance in nature. Environ. Microbiol..

[32] Macia M.D., Blanquer D., Togores B., Sauleda J., Perez J.L., Oliver A. (2005). Hypermutation Is a Key Factor in Development of Multiple-Antimicrobial Resistance in Pseudomonas aeruginosa Strains Causing Chronic Lung Infections. Antimicrob. Agents Chemother..

[33] Sköld O. (2000). Sulfonamide resistance: Mechanisms and trends. Drug Resist. Updates.

[34] Lupo A., Coyne S., Berendonk T. (2012). Origin and evolution of antibiotic resistance: The common mechanisms of emergence and spread in water bodies. Front. Microbiol..

[35] Goossens H., Ferech M., Vander Stichele R., Elseviers M. (2005). Outpatient antibiotic use in Europe and association with resistance: A cross-national database study. Lancet.

[36] Bartlett J.G., Gilbert D.N., Spellberg B. (2013). Seven ways to preserve the miracle of antibiotics. Clin. Infect. Dis..

[37] Knapp C.W., Dolfing J., Ehlert P.A.I., Graham D.W. (2010). Evidence of Increasing Antibiotic Resistance Gene Abundances in Archived Soils since 1940. Environ. Sci. Technol..

[38] Newman D., Cragg G. (2007). Natural Products as Sources of New Drugs over the Last 25 Years. J. Nat. Prod..

[39] Hatosy S.M., Martiny A.C. (2015). The ocean as a global reservoir of antibiotic resistance genes. Appl. Environ. Microbiol..

[40] O’Neill J. (2016). The Review on Antimicrobial Resistance. Tackling Drug-Resistant Infections Globally: Final Report and Recommendations.

[41] Kasanah N., Hamann M.T. (2004). Development of antibiotics and the future of marine microorganisms to stem the tide of antibiotic resistance. Curr. Opin. Investig. Drugs.

[42] Ventola C.L. (2015). The antibiotic resistance crisis: Part 1: Causes and threats. Pharm. Ther..

[43] Holmes A.H., Moore L.S.P., Sundsfjord A., Steinbakk M., Regmi S., Karkey A., Guerin P.J., Piddock L.J.V. (2016). Understanding the mechanisms and drivers of antimicrobial resistance. Lancet.

[44] Waglechner N., Wright G.D. (2017). Antibiotic resistance: It’s bad, but why isn’t it worse?. BMC Biol..

[45] Newman D.J., Cragg G.M. (2020). Natural Products as Sources of New Drugs over the Nearly Four Decades from 01/1981 to 09/2019. J. Nat. Prod..

[46] Newman D., Cragg G. (2016). Natural Products as Sources of New Drugs from 1981 to 2014. J. Nat. Prod..

[47] Henkel T., Brunne R.M., Müller H., Reichel F. (1999). Statistical Investigation into the Structural Complementarity of Natural Products and Synthetic Compounds. Angew. Chem. Int. Ed..

[48] Feher M., Schmidt J.M. (2003). Property distributions: Differences between drugs, natural products, and molecules from combinatorial chemistry. J. Chem. Inf. Comput. Sci..

[49] Evans B.E., Rittle K.E., Bock M.G., Dipardo R.M., Freidinger R.M., Whitter W.L., Lundell G.F., Veber D.F., Anderson P.S., Chang R.S. (1988). Methods for drug discovery: Development of potent, selective, orally effective cholecystokinin antagonists. J. Med. Chem..

[50] Welsch M.E., Snyder S.A., Stockwell B.R. (2010). Privileged scaffolds for library design and drug discovery. Curr. Opin. Chem. Biol..

[51] Breinbauer R., Vetter I.R., Waldmann H. (2002). From protein domains to drug candidates—Natural products as guiding principles in the design and synthesis of compound libraries. Angew. Chem. Int. Ed..

[52] Baltz R.H. (2008). Renaissance in antibacterial discovery from actinomycetes. Curr. Opin. Pharmacol..

[53] Ganesan A. (2008). The impact of natural products upon modern drug discovery. Curr. Opin. Chem. Biol..

[54] Lipinski C.A., Lombardo F., Dominy B.W., Feeney P.J. (1997). Experimental and computational approaches to estimate solubility and permeability in drug discovery and development settings. Adv. Drug Deliv. Rev..

[55] Koehn F., Carter G. (2005). The evolving role of natural products in drug discovery. Nat. Rev. Drug Discov..

[56] Baker D.D., Chu M., Oza U., Rajgarhia V. (2007). The value of natural products to future pharmaceutical discovery. Nat. Prod. Rep..

[57] János B. (2005). Bioactive Microbial Metabolites. J. Antibiot..

[58] Payne D.J., Gwynn M.N., Holmes D.J., Pompliano D.L. (2006). Drugs for bad bugs: Confronting the challenges of antibacterial discovery. Nat. Rev. Drug Discov..

[59] Newman D.J., Cragg G.M., Battershill C.N. (2009). Therapeutic agents from the sea: Biodiversity, chemo-evolutionary insight and advances to the end of Darwin’s 200th year. Diving Hyperb. Med..

[60] Schinke C., Martins T., Queiroz S.C.N., Melo I.S., Reyes F.G.R. (2017). Antibacterial Compounds from Marine Bacteria, 2010–2015. J. Nat. Prod..

[61] Kong D.X., Jiang Y.Y., Zhang H.Y. (2010). Marine natural products as sources of novel scaffolds: Achievement and concern. Drug Discov. Today.

[62] Jose P.A., Jha B. (2016). New Dimensions of Research on Actinomycetes: Quest for Next Generation Antibiotics. Front. Microbiol..

[63] Zhang G., Li J., Zhu T., Gu Q., Li D. (2016). Advanced tools in marine natural drug discovery. Curr. Opin. Biotechnol..

[64] Covington B.C., Xu F., Seyedsayamdost M.R. (2021). A Natural Product Chemist’s Guide to Unlocking Silent Biosynthetic Gene Clusters. Annu. Rev. Biochem..

[65] Luo Y., Cobb R.E., Zhao H. (2014). Recent advances in natural product discovery. Curr. Opin. Biotechnol..

[66] Lewis W.H., Tahon G., Geesink P., Sousa D.Z., Ettema T.J.G. (2021). Innovations to culturing the uncultured microbial majority. Nat. Rev. Microbiol..

[67] Zhang L., An R., Wang J., Sun N., Zhang S., Hu J., Kuai J. (2005). Exploring novel bioactive compounds from marine microbes. Curr. Opin. Microbiol..

[68] Dührkop K., Fleischauer M., Ludwig M., Aksenov A.A., Melnik A.V., Meusel M., Dorrestein P.C., Rousu J., Böcker S. (2019). SIRIUS 4: A rapid tool for turning tandem mass spectra into metabolite structure information. Nat. Methods.

[69] Guijas C., Montenegro-Burke J.R., Domingo-Almenara X., Palermo A., Warth B., Hermann G., Koellensperger G., Huan T., Uritboonthai W., Aisporna A.E. (2018). METLIN: A Technology Platform for Identifying Knowns and Unknowns. Anal. Chem..

[70] Wang M., Carver J.J., Phelan V.V., Sanchez L.M., Garg N., Peng Y., Nguyen D.D., Watrous J., Kapono C.A., Luzzatto-Knaan T. (2016). Sharing and community curation of mass spectrometry data with Global Natural Products Social Molecular Networking. Nat. Biotechnol..

[71] Medema M.H., Blin K., Cimermancic P., de Jager V., Zakrzewski P., Fischbach M.A., Weber T., Takano E., Breitling R. (2011). antiSMASH: Rapid identification, annotation and analysis of secondary metabolite biosynthesis gene clusters in bacterial and fungal genome sequences. Nucleic Acids Res..

[72] Ling L.L., Schneider T., Peoples A.J., Spoering A.L., Engels I., Conlon B.P., Mueller A., Schäberle T.F., Hughes D.E., Epstein S. (2015). A new antibiotic kills pathogens without detectable resistance. Nature.

[73] Wright G. (2015). Antibiotics: An irresistible newcomer. Nature.

[74] McCarthy M.W. (2019). Teixobactin: A novel anti-infective agent. Expert Rev. Anti-Infect. Ther..

[75] Lebar M.D., Heimbegner J.L., Baker B.J. (2007). Cold-water marine natural products. Nat. Prod. Rep..

[76] Skropeta D. (2008). Deep-sea natural products. Nat. Prod. Rep..

[77] Challinor V.L., Bode H.B. (2015). Bioactive natural products from novel microbial sources. Ann. N. Y. Acad. Sci..

[78] Barka E.A., Vatsa P., Sanchez L., Gaveau-Vaillant N., Jacquard C., Klenk H.P., Clement C., Ouhdouch Y., Wezel G.P.V. (2016). Taxonomy, Physiology, and Natural Products of Actinobacteria. Microbiol. Mol. Biol. Rev..

[79] Watve M., Tickoo R., Jog M., Bhole B. (2001). How many antibiotics are produced by the genus Streptomyces?. Arch. Microbiol..

[80] Nett M., Ikeda H., Moore B.S. (2009). Genomic basis for natural product biosynthetic diversity in the actinomycetes. Nat. Prod. Rep..

[81] William F., Paul R.J. (2006). Developing a new resource for drug discovery: Marine actinomycete bacteria. Nat. Chem. Biol..

[82] Gaspari F., Paitan Y., Mainini M., Losi D., Ron E.Z., Marinelli F. (2005). Myxobacteria isolated in Israel as potential source of new anti-infectives. J. Appl. Microbiol..

[83] Davila-Cespedes A., Hufendiek P., Crusemann M., Schaberle T., Konig G. (2016). Marine-derived myxobacteria of the suborder Nannocystineae: An underexplored source of structurally intriguing and biologically active metabolites. Beilstein J. Org. Chem..

[84] Gemperlein K., Zaburannyi N., Garcia R., La Clair J., Müller R. (2018). Metabolic and Biosynthetic Diversity in Marine Myxobacteria. Mar. Drugs.

[85] Schäberle T.F., Goralski E., Neu E., Erol O., Hölzl G., Dörmann P., Bierbaum G., König G.M. (2010). Marine myxobacteria as a source of antibiotics--comparison of physiology, polyketide-type genes and antibiotic production of three new isolates of Enhygromyxa salina. Mar. Drugs.

[86] Schäberle T.F., Lohr F., Schmitz A., König G.M. (2014). Antibiotics from myxobacteria. Nat. Prod. Rep..

[87] Nunnery J.K., Mevers E., Gerwick W.H. (2010). Biologically active secondary metabolites from marine cyanobacteria. Curr. Opin. Biotechnol..

[88] Pham J.V., Yilma M.A., Feliz A., Majid M.T., Maffetone N., Walker J.R., Kim E., Cho H.J., Reynolds J.M., Song M.C. (2019). A Review of the Microbial Production of Bioactive Natural Products and Biologics. Front. Microbiol..

[89] Hu Y., Chen J., Hu G., Yu J., Zhu X., Lin Y., Chen S., Yuan J., Taglialatela-Scafati O. (2015). Statistical Research on the Bioactivity of New Marine Natural Products Discovered during the 28 Years from 1985 to 2012. Mar. Drugs.

[90] Donadio S., Monciardini P., Sosio M. (2007). Polyketide synthases and nonribosomal peptide synthetases: The emerging view from bacterial genomics. Nat. Prod. Rep..

[91] Belknap K.C., Park C.J., Barth B.M., Andam C.P. (2020). Genome mining of biosynthetic and chemotherapeutic gene clusters in Streptomyces bacteria. Sci. Rep..

[92] Rath C.M., Janto B., Earl J., Ahmed A., Hu F.Z., Hiller L., Dahlgren M., Kreft R., Yu F., Wolff J.J. (2011). Meta-omic characterization of the marine invertebrate microbial consortium that produces the chemotherapeutic natural product ET-743. ACS Chem. Biol..

[93] Lago J., Rodríguez L.P., Blanco L., Vieites J.M., Cabado A.G. (2015). Tetrodotoxin, an Extremely Potent Marine Neurotoxin: Distribution, Toxicity, Origin and Therapeutical Uses. Mar. Drugs.

[94] Kristoffersen V., Rämä T., Isaksson J., Andersen J.H., Gerwick W.H., Hansen E. (2018). Characterization of Rhamnolipids Produced by an Arctic Marine Bacterium from the *Pseudomonas fluorescence* Group. Mar. Drugs.

